# Flexible Porous Pomelo Pith Derived Janus Liquid Metal and Adhesive Hydrogel Hybrid Electronic Skins

**DOI:** 10.1002/smmd.70040

**Published:** 2026-06-28

**Authors:** Jinbo Li, Wenzhao Li, Hongbo Zhang, Yi Yang, Yuanjin Zhao

**Affiliations:** ^1^ Department of Cardiovascular Surgery Ruijin Hospital Shanghai Jiao Tong University School of Medicine Shanghai China; ^2^ Wenzhou Institute University of Chinese Academy of Sciences Wenzhou China; ^3^ Pharmaceutical Sciences Laboratory Åbo Akademi University Turku Finland; ^4^ Turku Bioscience Centre University of Turku and Åbo Akademi University Turku Finland; ^5^ Department of Rheumatology and Immunology Nanjing Drum Tower Hospital School of Biological Science and Medical Engineering Southeast University Nanjing China

**Keywords:** adhesive, electronic skin, hydrogel, liquid metal, pomelo pith

## Abstract

Liquid metal (LM)‐derived electronic skin (e‐skin) exhibits significant potential in the domains of human‐machine interfaces, health monitoring and energy management. Herein, we introduce a cost‐effective paradigm of LMs‐derived e‐skin featuring anti‐freezing capability, efficient thermal management, and motion detection. The inexpensive and eco‐friendly porous pomelo pith (PP) is employed as the substrate for the patterning of LM and as the physical scaffold for adhesive hydrogels. The unique porous architecture of the PP provides a natural template for the integration of LM and hydrogels, which facilitates the creation of electronic devices with improved flexibility and adhesion. The diverse patterning of nickel (Ni)‐doped LM on the rough and porous pomelo pith surface is facilitated by the attractive force of a magnetic field applied below. The adhesive pre‐gel is readily drawn into the pores of the pomelo pith due to capillary action. The resulting LM/PP hydrogel electronics are capable of rapid and stable Joule heating behavior and reliable motion detection. Additionally, the incorporation of glycerol endows this e‐skin with superior freezing and desiccation resistance. Thus, this facile fabrication strategy successfully integrates excellent conductivity, adhesion, and environmental resilience, offering a promising paradigm for advanced wearable electronics.

## Key Points

- The unique porous architecture of eco‐friendly pomelo pith provides a natural template for the integration of liquid metal and hydrogel.
- The incorporation of glycerol endows this e‐skin with freezing and desiccation resistance.
- The LM/PP hydrogel e‐skin is capable of rapid and stable Joule heating behavior and reliable motion detection.

## Graphical abstract

We introduce a cost‐effective paradigm of LM‐derived e‐skin featuring anti‐freezing capability, efficient thermal management, and motion detection. The eco‐friendly porous pomelo pith (PP) is employed as the substrate for the patterning of LM and as the physical scaffold for adhesive hydrogels.

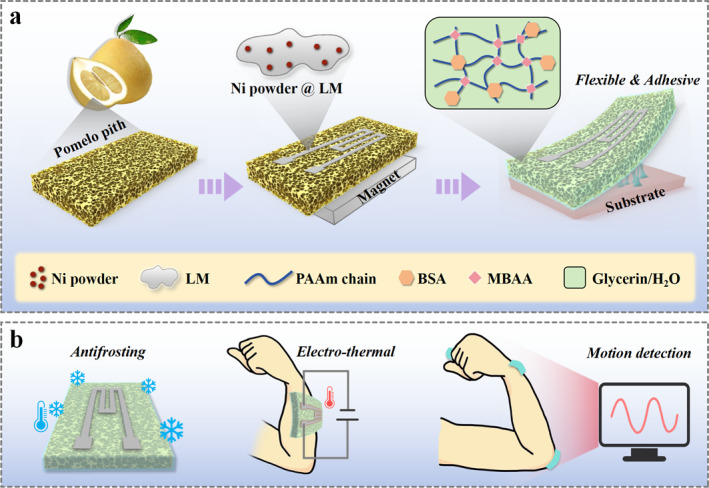

## 1 Introduction

Electronic skin (e‐skin) is a multifunctional electronic device fabricated from novel functional materials, which are flexible, stretchable and capable of seamless conformation to human skin [1, 2]. It holds promising prospects in human‐machine interfaces, health monitoring and energy management [3, 4, 5]. The need for e‐skin to effectively interact with and function at the biological interface imposes strict requirements on its electrical and adhesive properties [6, 7]. Among various conductive materials used in e‐skin, gallium (Ga)‐based liquid metals (LMs) have demonstrated unique advantages due to their superior structural flexibility, electrical conductivity and biocompatibility [8, 9, 10, 11, 12]. However, their fluidic state makes them prone to leakage, posing a critical challenge for safe and stable operation [13, 14]. To address this, various encapsulation strategies have been developed, such as microchannel encapsulation, core–shell microencapsulation and matrix dispersion encapsulation [15, 16, 17, 18, 19]. Despite their great potential, they often involve complex fabrication processes, high costs, and demanding technical requirements. In addition, achieving robust and long‐term adhesion to dynamic and irregular surfaces, particularly the human skin, remains a significant obstacle. Therefore, developing new types of LM‐based e‐skin systems that are simpler to fabricate, cost‐effective, and capable of reliable skin adhesion continues to be a highly promising and active field of research.

**FIGURE 1:**
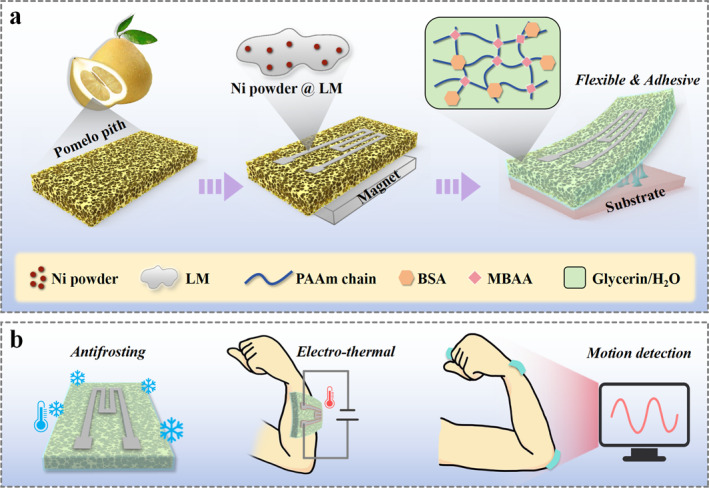
The scheme of LM/PP hydrogel e‐skin. (a) The flexible device is constructed with liquid metal circuitry and porous pomelo pith scaffold, which is infiltrated with adhesive hydrogel. (b) The antifrosting LM/PP hydrogel e‐skin is applied for motion detection and efficient thermal management.

Herein, we propose a new paradigm of e‐skin featuring anti‐freezing capability, efficient thermal management, and motion detection based on porous pomelo pith, as shown in Figure 1. Using porous scaffolds to integrate conductive and adhesive materials is an effective strategy for e‐skin design, for which the sponge layer of pomelo pith serves as a readily available natural candidate. Its unique porous structure is employed as the substrate for the patterning of LM and as the physical scaffold for adhesive hydrogels. The techniques to shape and pattern conductive circuits have been an indispensable part of manufacturing functional electronic devices [20, 21]. Given its inherent formability, LM represents an excellent material choice [22, 23, 24, 25, 26]. Moreover, the adaptability and adhesion to rough surfaces pose critical requirements for the development of hydrogel‐based electronic devices, particularly in scenarios where their interfaces directly interact with biological tissues or are subjected to mechanical stress [27, 28, 29]. Developing hydrogels with fortified mechanical strength and reliable adhesive properties is crucial for the durability and performance of flexible electronic devices in real‐world settings.

In this paper, the desired flexible device was fabricated utilizing LM circuitry and a porous pomelo pith (PP) scaffold, which was infiltrated with an adhesive hydrogel. The adhesive pre‐gel is composed of acrylamide, bovine serum albumin (BSA) and glycerol. The adhesive property is enhanced by incorporating BSA into the acrylamide hydrogel matrix. In our method, the patterning of nickel (Ni)‐doped LM on the rough and porous pomelo pith surface is facilitated by the attractive force of a magnetic field applied from below. Subsequently, the adhesive pre‐gel is readily drawn into the pores of the pomelo pith due to capillary action. The resulting LM/PP hydrogel e‐skin demonstrates reliable performance in sensitive human motion detection and efficient thermal management. We have also verified the cytocompatibility and hemocompatibility of this LM/PP hydrogel e‐skin in vitro. In addition, the strategic incorporation of glycerol into the hydrogel matrix endows the e‐skin with freeze and desiccation resistance, which is advantageous for operation under harsh environmental conditions. Thus, this facile fabrication strategy successfully integrates conductive, adhesive, and environmentally‐resilient properties and offers a promising paradigm for advanced wearable electronics.

## 2 Results and Discussion

### 2.1 Pretreatment of Pomelo Pith and Patterning of LM

**FIGURE 2:**
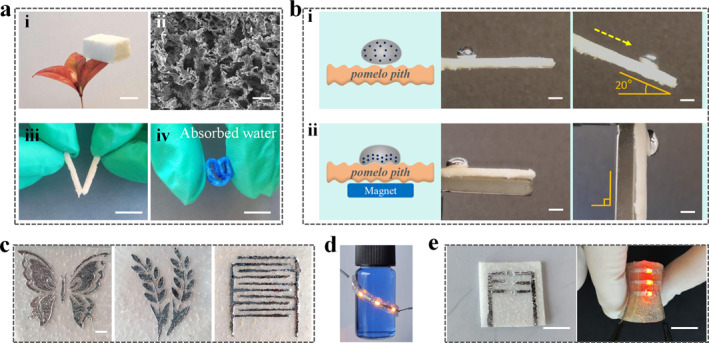
The pretreatment of pomelo pith and patterning of LM. (a) Morphology of pomelo pith. (i) The processed pomelo pith is very light in weight and will not weigh down the fragile leaves. (ii) The porous microstructure of the pomelo pith. (iii) The dried pomelo pith is brittle. (iv) The water‐absorbing pomelo pith is soft. (b) Droplet adhesion on the PP substrate: (i) without a magnetic field and (ii) under a magnetic field. (c) Patterned PP with butterfly, wheat, and interdigitated electrode shapes. (d) Patterned LM/PP hydrogel e‐skin with a series circuit. (e) Patterned LM/PP hydrogel e‐skin with a parallel circuit. Scale bars: a (i, iii, iv) = 1 cm, a (ii) = 200 μm, b, c = 1 mm, e = 1 cm.

For a specific type of material with highly porous, honeycomb‐like structures, we selected the pith of the pomelo peel, a sponge‐like layer that accounts for the primary thickness of the rind, as the raw material [30]. After undergoing alkali treatment, the PP was effectively delignified and depectinated, yielding a loose and porous skeletal structure composed predominantly of cellulose (Figure 2ai,ii). Upon water absorption, the brittle dry PP became soft, indicating the potential for bending through the imbibition of aqueous solutions (Figure 2aiii,iv). Furthermore, the rough surface of the PP offered excellent attachment sites for the conductive pattern and the addition of Ni microparticles increased the adhesion. The gravity alone was not enough to support the droplet adhesion (Figure 2bi). However, upon the introduction of the magnetic field, the adhesion force was enhanced as Ni microparticles aggregated, leading to an increased contact between the droplet and the PP substrate (Figure 2bii) [31, 32, 33]. Consequently, leveraging the aforementioned magnetic field effect, we performed scrape‐coating with masks on the rough surface of PP, allowing the slurry to be simultaneously subjected to both magnetic attraction and mechanical pressure. This approach thereby successfully printed elaborate patterns, including butterfly, wheat, and interdigitated electrode configurations (Figure 2c).

Furthermore, owing to the above superior patternability, we printed multi‐unit series (Figure 2d) and parallel (Figure 2e) circuits using surface‐mounted device LEDs as electrical loads. Then, we filled the porous structure of the patterned PP with adhesive pre‐gel. Driven by capillary forces, the pre‐gel penetrated into the porous structure of the PP, and the LM/PP hydrogel e‐skin was subsequently fabricated via a photopolymerization process. The adhesive pre‐gel is composed of acrylamide, BSA and glycerol. The resulting LM/PP hydrogel e‐skin could adhere to glass bottles, and the LEDs remained illuminated during the bending process, demonstrating the feasibility of using the combination of LM patterned PP and adhesive hydrogels to fabricate e‐skin. The PP hydrogel with S‐shaped conductive pathway could still rely on the adhesion of the hydrogel and the fluidity of LM to relight the lamp after being cut, demonstrating its ability to withstand mechanical damage (Supporting Information S1: Figure S1).

### 2.2 Adhesion Property

**FIGURE 3:**
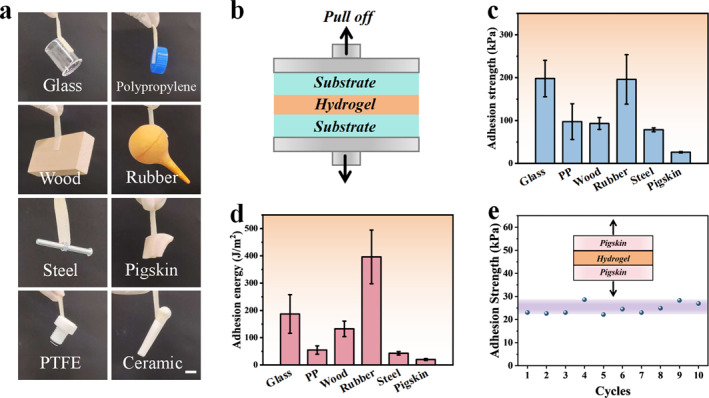
The adhesion properties of LM/PP hydrogel e‐skin. (a) Pictures showing the adhesion to glass, polypropylene, wood, rubber, steel, pigskin, PTFE and ceramic. (b) Diagram of the adhesion mechanism. (c) Adhesion strength of the LM/PP hydrogel. (d) Adhesion energy of the LM/PP hydrogel. (e) Cyclic adhesion and detachment experiments of the LM/PP hydrogel from pigskin. Scale bar: 1 cm.

The interfacial adhesion of the LM/PP hydrogel e‐skin was also investigated. The resulting hydrogel could firmly adhere to different substrates including glass, polypropylene, wood, rubber, steel, pigskin, poly(tetrafluoroethylene) (PTFE), ceramic (Figure 3a), indicating its potential for versatile sensor deployment, such as on the surfaces of intelligent robots. To quantitatively assess the interfacial adhesive behavior, the LM/PP hydrogel was sandwiched between two substrates (Figure 3b), and the corresponding adhesion energy and adhesion strength were measured (Supporting Information S1: Figure S2a, Figure 3c,d). The adhesive strength on pigskin was approximately 1200 times the weight of the LM/PP hydrogel itself, which sufficient for practical applications. Notably, the PP hydrogel maintained stable adhesion performance on pigskin over at least 10 attachment–detachment cycles (Supporting Information S1: Figure S2b,c, Figure 3e). This robust cyclic adhesion endowed the e‐skin with good repositionability and reusability. Furthermore, the PP hydrogel could conformally adhere to human finger joints (Supporting Information S1: Figure S3b). Upon removal from the hand, comb‐like stretching could be observed leaving no discernible remnants on the skin (Supporting Information S1: Figure S3c), ensuring the skin surface cleanliness.

### 2.3 Mechanical and Electrical Properties

**FIGURE 4:**
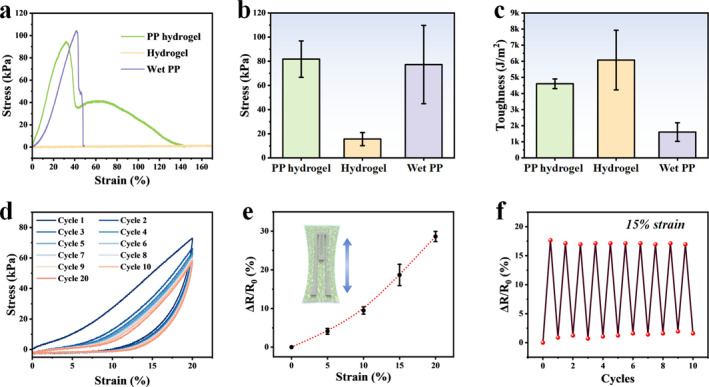
The mechanical and electrical properties of LM/PP hydrogel e‐skin. (a) Uniaxial tensile curves of the PP hydrogel, hydrogel without PP, and wet PP. (b) Maximum stress of the PP hydrogel, hydrogel without PP, and wet PP. (c) Toughness of the PP hydrogel, hydrogel without PP, and wet PP. (d) Uniaxial tensile curves of the PP hydrogel over 1 to 20 loading/unloading cycles. (e) The resistance response under strain of LM/PP e‐skin. (f) Relative resistance change of LM‐patterned PP hydrogel over 10 stretching cycles.

Through the comparative analysis of the tensile mechanics of pure hydrogel, wet PP and PP hydrogel, it could be seen that the PP scaffold endowed the PP hydrogel with good stress‐bearing capacity (Figure 4a,b). Meanwhile, the incorporation of the hydrogel network enabled the PP hydrogel to exhibit enhanced toughness due to effective energy dissipation (Figure 4c). After conducting 20 consecutive tensile‐recovery cycle tests on the PP hydrogel, it was found that starting from the second cycle, the stress‐strain curve changed slightly, and the hysteresis area remained stable, indicating that the material possesses good elastic recovery ability and cyclic stability (Figure 4d). The above mechanical data suggested that the integration of PP and adhesive hydrogel effectively enhanced the overall mechanical properties, enabling PP hydrogel to satisfy the application requirements of e‐skin. In addition, the electrical properties of the LM/PP hydrogel e‐skin with conventional serpentine pattern were tested. The results revealed that during the stretching deformation, the relative change rate of resistance of the material continuously increased with strain, demonstrating good strain‐response characteristics (Figure 4e). After 10 cyclic stretching tests, the resistance change rate remained stable (Figure 4f), demonstrating that the LM/PP hydrogel e‐skin still had good electrical conductivity stability and repeatability under dynamic deformation conditions, showing its application potential in stretchable electronic devices and wearable strain sensors.

### 2.4 Biocompatibility

**FIGURE 5:**
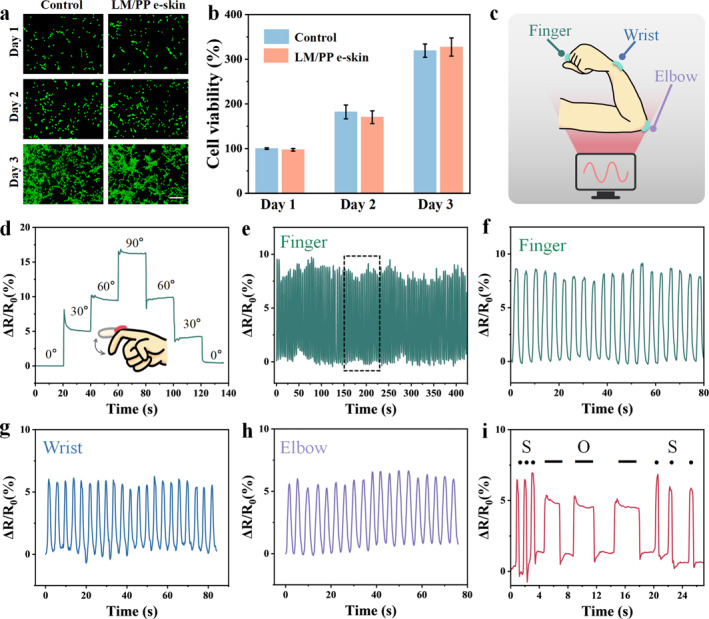
(a) Live/dead assay of NIH‐3T3 cells. (b) Cell viability in the CCK‐8 test. (c) Schematic diagram of monitoring human motion signals. (d) Different angles of finger bending. (e, f) Repeated finger bending. (g) Repeated wrist bending. (h) Repeated elbow bending. (i) The Morse code “SOS.” Scale bar: 200 μm.

Hydrogel‐based electronic devices often come into contact with the human body, so their biocompatibility is of particular importance. The LM, natural pomelo pith, and hydrogel matrix in LM/PP hydrogel e‐skin were generally recognized as biocompatible materials. The cell biocompatibility assessments utilized NIH‐3T3 fibroblasts as the model cell line. Live/dead cell staining demonstrated that the NIH‐3T3 cells in the LM/PP e‐skin group and the control group (without treatment) all exhibited good proliferation and normal morphology (Figure 5a). The quantitative results of the Cell Counting Kit‐8 (CCK‐8) further indicated that within 3 days, the cell viability of LM/PP e‐skin group did not decline compared to the control group (Figure 5b). Furthermore, hemocompatibility was examined via an in vitro hemolysis assay. No significant hemolysis was observed upon contact with the LM/PP e‐skin, indicating the safety of its interaction with blood components (Supporting Information S1: Figure S3a).

### 2.5 LM/PP Hydrogel e‐Skin Used as the Strain Sensor

The LM/PP hydrogel e‐skin exhibited good adhesion, fatigue resistance, and electrical properties. Consequently, it was employed as a strain sensor that could conformably attach to human joints to detect various motion signals (Figure 5c, Supporting Information S1: Figure S3d). When the finger maintained four fixed angles of 0°, 30°, 60°, and 90°, the relative resistance signals remained basically stable (Figure 5d). Simultaneously, the sensor exhibited a rapid response to stepwise deformation, effectively tracking the specific bending trends. The resistance value exhibited periodic variations when the fingers were rapidly bent for more than 100 cycles (Figure 5e,f). This e‐skin also demonstrated reliable sensing performance for wrist and elbow motion monitoring (Figure 5g,h). In addition, the sensor could output Morse codes such as “SOS” for signal transmission and emergency calls (Figure 5i). The dynamic signals corresponding to joint activities can be further exploited for encoding and decoding, demonstrating the potential in human‐machine interaction.

### 2.6 The Electrothermal and Environmental Tolerance

**FIGURE 6:**
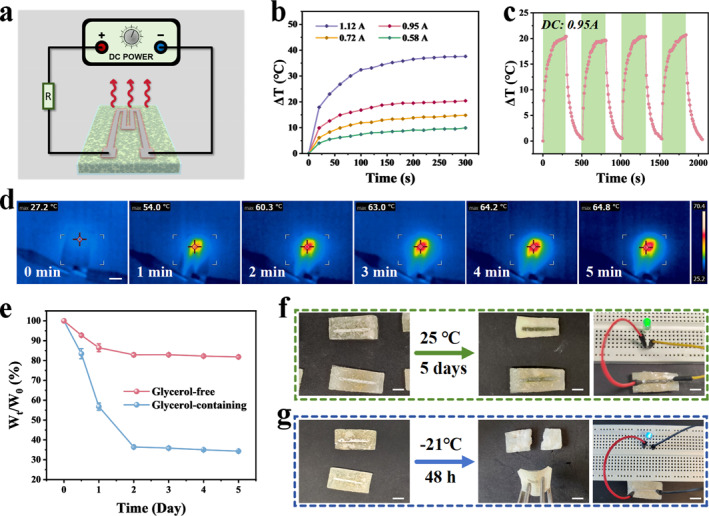
(a) Schematic diagram of the Joule heating behavior of LM/PP hydrogel e‐skin. (b) Record of temperature increment of LM/PP hydrogel e‐skin over time under different constant currents. (c) The temperature increase variation of LM/PP hydrogel e‐skin during 4 consecutive on‐off cycles. (d) Thermal image under the current of 1.12 A. (e) Water retention of LM/PP hydrogel e‐skin with and without glycerol over 5 days. (f) Appearance of LM/PP hydrogel e‐skin with (lower) and without (upper) glycerol after storage at room temperature (25°C) for 5 days. (g) Appearance of LM/PP hydrogel e‐skin with (lower) and without (upper) glycerol after storage at −21°C for 48 h. Scale bars: 5 mm.

The LM/PP hydrogel e‐skin also demonstrated remarkable potential in thermal management and adaptability to complex environments. Under application of a DC current, the LM circuit exhibited significant Joule heating effects and the surface temperature of the e‐skin could be elevated rapidly. The representative heater was fabricated using the LM/PP hydrogel e‐skin (Figure 6a). Under the loading of a constant current, the temperature rose rapidly (Figure 6b,d). Moreover, this heater exhibited stable heating performance over 4 consecutive switching drive cycles, as shown in Figure 6c. This rapid and stable Joule heating behavior indicates that the LM/PP hydrogel e‐skin holds great promise for applications in wearable heaters and de‐icing systems. Additionally, owing to the excellent capability of hydrogels as drug carriers, the LM/PP hydrogel e‐skin may offer opportunities for electrothermally triggered drug release after drug loading.

Furthermore, we evaluated the anti‐freezing and anti‐desiccation capabilities of the LM/PP hydrogel e‐skin under simulated extreme environments. These properties were mainly attributed to the incorporation of glycerol. As shown in Figure 6e, the glycerol‐containing e‐skin exhibited remarkably enhanced water retention, while the glycerol‐free e‐skin underwent rapid dehydration. This enhancement can be ascribed to the hygroscopic character of glycerol, which reduces water evaporation and sustains the hydrogel network stability by forming hydrogen bonds with water molecules. After 5 days, the glycerol‐free e‐skin showed clear signs of dehydration (Figure 6f). Meanwhile, the glycerol‐containing e‐skin retained its original appearance, highlighting its improved long‐term stability. The antifreeze performance was assessed by keeping the hydrogels at −21°C for 48 h. The glycerol‐free e‐skin became visibly hard and brittle. In contrast, the glycerol‐containing e‐skin remained soft and unfrozen (Figure 6g). This is likely due to the freezing point depression introduced by glycerol, which disrupts ice crystal formation within the hydrogel matrix. These findings collectively demonstrate that the e‐skin exhibits tolerance under extreme environments, benefiting significantly from the introduction of glycerol. Consequently, this material shows potential for practical applications in flexible and wearable electronic devices, providing stable performance under fluctuating temperature and humidity as well as during harsh transportation and storage conditions.

## 3 Conclusion and Prospect

In summary, this study demonstrates a novel approach to fabricate flexible electronic skin by integrating Ni‐doped Ga‐based LM and adhesive hydrogels onto the naturally porous structure of pomelo pith. The magnetic patterning of LM and capillary‐driven infusion of hydrogel into the PP scaffold result in a composite material with excellent flexibility, adhesion, and functionality. The LM/PP hydrogel e‐skin exhibits effective thermal management, sensitive human motion detection, and enhanced durability under extreme conditions due to glycerol incorporation. These findings highlight the potential of biomaterial‐based substrates and liquid metal technologies in advancing next‐generation wearable electronics and smart sensing systems.

Furthermore, the comprehensive performance of this e‐skin opens avenues for the management of cardiovascular diseases. Given its comfortable and long‐term conformal contact with the skin, this e‐skin holds promise for continuous, non‐invasive physiological monitoring, particularly for capturing occasional or paroxysmal arrhythmias via long‐term electrocardiography (ECG). Beyond diagnostics, the proposed platform is envisioned to enable localized and precise drug delivery by encapsulating therapeutic agents within the hydrogel matrix. Specifically, future studies may explore its use for conformal adhesion to critical lesion sites, such as bypass grafts during coronary artery bypass grafting (CABG) to prevent restenosis or the myocardium to mitigate adverse remodeling, thereby offering targeted strategies for cardiovascular therapy.

## 4 Methods

### 4.1 Pretreatment of Pomelo Pith

The exocarp of the fresh pomelo peel was removed, leaving the middle sponge layer. Then, cut the pomelo piths into rectangular small blocks for storage. Soak the pomelo piths in water and use negative pressure to allow the water to penetrate into the pores of the piths. Then, remove the excess water. Subsequently, soak the piths in a NaOH solution for 12 h, then bleach them with hydrogen peroxide for 12 h. Then, thoroughly wash them with water. Finally, the samples are freeze‐dried.

### 4.2 Preparation of LM/PP Hydrogel e‐Skin

The LM conductive filler was prepared by mixing eutectic gallium‐indium alloy (EGaIn) with nickel (Ni) powder. Specifically, the Ni powder and the EGaIn were sequentially added to a mortar and then blended with a pestle. Optionally, prior to infusion of the adhesive pre‐gel, the LM conductive filler was patterned onto the pretreated dried PP. For this process, a magnet was positioned beneath the PP, and a mask was fixed on top. The LM conductive filler was then uniformly spread across the mask, after which the mask was carefully removed to yield the desired pattern. The detailed preparation procedure of adhesive pre‐gel was as follows: albumin from bovine serum (BSA, 2.5 g), acrylamide (AAm, 3 g), 2‐hydroxy‐2‐methyl‐1‐phenyl‐1‐propanone (HMPP, 0.1 mL) and N,N′‐methylenebisacrylamide (MBAA, 0.15 mL of a 20 mg/mL stock solution) were introduced into 10 mL mixture of glycerol and water (1:1 by volume). After thorough mixing, a light yellow transparent pre‐gel was obtained. To prevent the leakage of the LM pattern, the top surface of the pretreated PP was encapsulated by a thin layer (0.5 mm) of a solidified hydrogel. Subsequently, the pre‐gel was injected into the pretreated PP and infiltrated the porous structure. Then, the PP hydrogel was prepared via a UV‐crosslinked process. Since BSA typically undergoes thermal denaturation above 70°C, an ice box was used during the crosslinking process to maintain a low reaction temperature and prevent protein denaturation.

### 4.3 Adhesion and Mechanical Testing

The adhesion between the hydrogels and a series of substrates was measured by a universal material testing machine (Instron, USA). For the probe pull test, substrates were first glued onto custom‐made parallel fixtures. The hydrogel samples (15 mm × 15 mm × 2 mm) were brought into contact with the substrates under a 10 N pre‐compression force. The adhesion was then measured by pulling the substrate off the hydrogel sample with a loading rate of 10 mm/min. For the uniaxial tensile tests, the samples were stretched from their original length until fracture with a loading rate of 10 mm/min. For the cyclic tests, the samples were cyclically stretched between 0% and 20% strain for a total of 20 cycles.

### 4.4 Electrical Testing and Strain Sensors

The resistance response under strain of the LM/PP e‐skin was measured by recording its electrical resistance with a digital multimeter (Keithley, USA) while the sample was stretched using a universal material testing machine. When the LM/PP e‐skin was utilized as a strain sensor to collect human motion signals, the same digital multimeter was employed to acquire the resistance.

### 4.5 In Vitro Cytocompatibility and Blood Compatibility

For the cytocompatibility, exponentially growing NIH‐3T3 fibroblasts were plated at a density of 5000 cells/well. Then, the control group was cultured with DMEM medium, and the experimental group was co‐cultured with the LM/PP hydrogel e‐skin. At predetermined time points (24, 48, 72 h), the CCK‐8 experiment and live/dead cell staining were conducted following the protocols provided by the manufacturers. To evaluate blood compatibility, fresh rat red blood cells (RBCs) were resuspended in PBS to obtain a 2% (v/v) suspension of RBCs. Then, the RBC suspension was incubated for 6 h at 37°C with the different material samples (PP, PP + LM, PP hydrogel, and LM/PP e‐skin). Subsequently, the samples were centrifuged (2500 rpm, 7 min), and the absorbances of the collected supernatants were measured at 540 nm to determine hemoglobin release. The positive and negative control groups consisted of RBCs exposed to deionized water and PBS, respectively.

### 4.6 Materials and Reagents

The liquid metal (75 wt % Ga and 25 wt % In) was supplied by Huatai Metal Materials Co. Ltd. The shatian pomelos were acquired from the local supermarket. The near‐spherical Ni microparticles (≈6 μm) were obtained from Guangzhou Metal & Metallurgy Co. Ltd. BSA (low fatty acid) was purchased from Macklin Inc. AAm, MBAA and HMPP were purchased from Aladdin Inc. Glycerol was supplied by Shanghai Hushi Chemical Co. Ltd. The CCK‐8 was obtained from Beyotime Biotechnology.

## Author Contributions

Y.Z. and H.Z. conceived the idea and designed the experiment. J.L. and W.L. conducted the experiments, analyzed the data, and wrote the manuscript. Y.Y. participated in the discussion of results. All authors were engaged in the scientific discussion, reviewed, and approved the final manuscript.

## Ethics Statement

The rat blood samples were archived surplus specimens obtained from a previously approved animal protocol by the Institutional Animal Care and Use Committee of WIUCAS (WIUCAS24101703). No additional live animal procedures were performed for this study.

## Conflicts of Interest

Hongbo Zhang is an executive editor for *Smart Medicine* and was not involved in the editorial review or the decision to publish this article. All authors declare that there are no competing interests.

## Supporting information


Supporting Information S1

## Data Availability

The data that support the findings of this study are available from the corresponding author upon reasonable request.
