# Supplementary material for: Flexible Porous Pomelo Pith Derived Janus Liquid Metal and Adhesive Hydrogel Hybrid Electronic Skins
Source: Smart Med. 2026 Jun 28;5(3):e70040. doi: 10.1002/smmd.70040 (PMC13317696; doi:10.1002/smmd.70040)
Supplement: Supplementary file 1 — Supporting Information S1 [file SMMD-5-e70040-s001.docx]

**Supplementary Information**

**Flexible porous pomelo pith derived Janus liquid metal and adhesive hydrogel hybrid electronic skins**

Jinbo Li ^1,2,3^, Wenzhao Li ^1,2^, Hongbo Zhang ^3,4,^*, Yi Yang ^1,^*, Yuanjin Zhao ^1,2,5,^*

^1^ Department of Cardiovascular Surgery, Ruijin Hospital, Shanghai Jiao Tong University School of Medicine, Shanghai 200025, China

^2^ Wenzhou Institute, University of Chinese Academy of Sciences, Wenzhou 325001, China

^3^ Pharmaceutical Sciences Laboratory, Faculty of Science and Engineering, Åbo Akademi University, Turku 20520, Finland

^4^ Turku Bioscience Centre, University of Turku and Åbo Akademi University, Turku 20520, Finland

^5^ Department of Rheumatology and Immunology, Nanjing Drum Tower Hospital, School of Biological Science and Medical Engineering, Southeast University, Nanjing 210096, China

Email: hongbo.zhang@abo.fi (H.B.Z.); yy12181@rjh.com.cn (Y.Y.);  yjzhao@seu.edu.cn (Y.J.Z.)


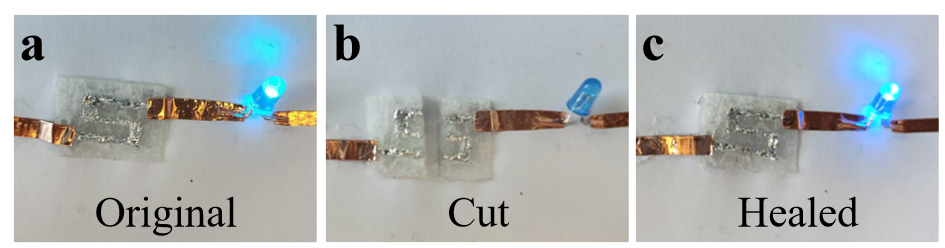


**Figure S1.** The LM/PP hydrogel with an S-shaped conductive pathway restored lamp illumination after cutting.


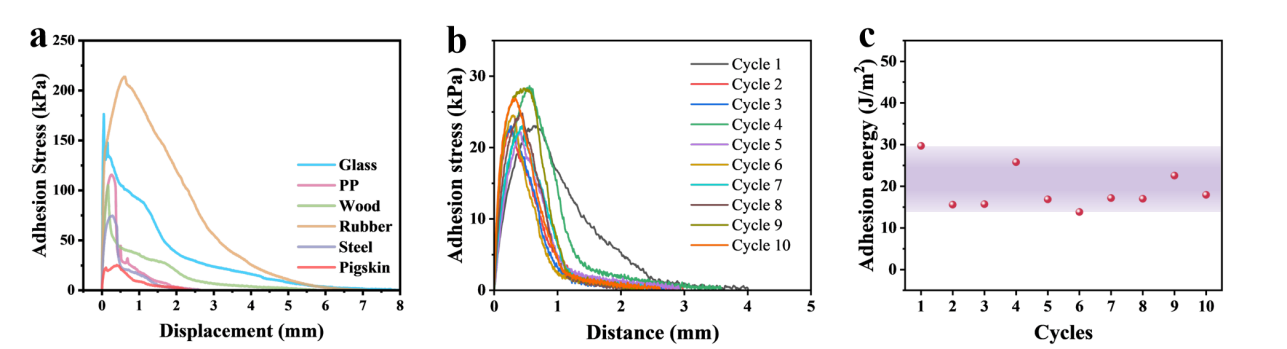


**Figure S2.** (a) Adhesion stress-displacement curves of the PP hydrogel on various substrates. (b) Cyclic adhesion performance of the PP hydrogel on pigskin over 10 repeated attachment-detachment cycles. (c) Adhesion energy of the PP hydrogel on pigskin over 10 cycles.


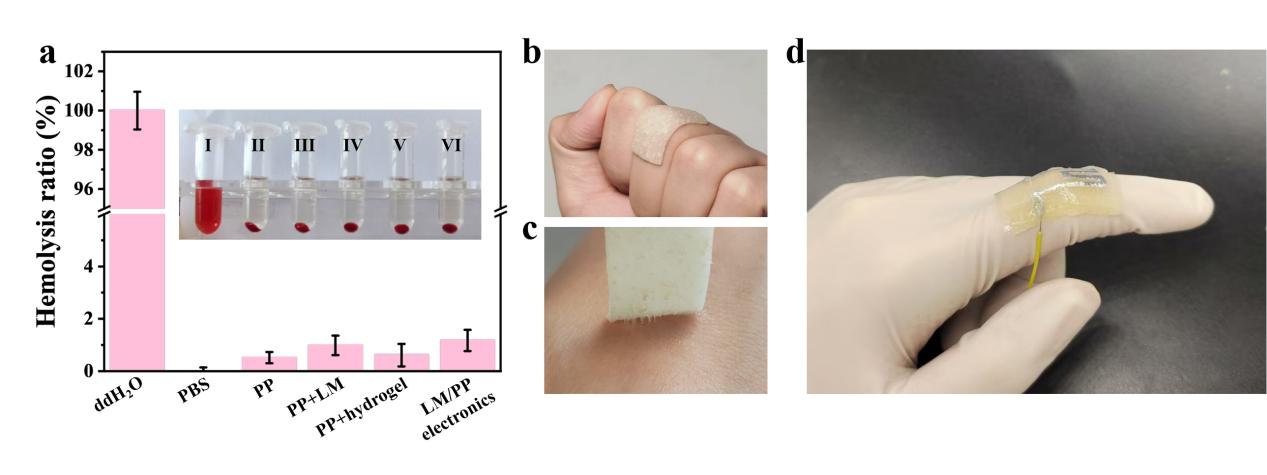


**Figure S3.** (a) Hemolysis test. (b) The LM/PP hydrogel adhered to the finger joint. (c) The LM/PP hydrogel peeled off from the back of the hand. (d) The LM/PP e-skin attached to a finger.
